# Membrane protein mediated bilayer communication in networks of droplet interface bilayers

**DOI:** 10.1038/s42004-020-0322-1

**Published:** 2020-06-12

**Authors:** Stuart Haylock, Mark S. Friddin, James W. Hindley, Enrique Rodriguez, Kalypso Charalambous, Paula J. Booth, Laura M. C. Barter, Oscar Ces

**Affiliations:** 1 Department of Chemistry, Molecular Sciences Research Hub, Imperial College London, 80 Wood Lane, London, W12 0BZ UK; 2 Institute of Chemical Biology, Molecular Sciences Research Hub, Imperial College London, 80 Wood Lane, London, W12 0BZ UK; 3 fabriCELL, Imperial College London, 80 Wood Lane, London, W12 0BZ UK; 4 Department of Chemistry, King’s College London, Britannia House, 7 Trinity Street, London, SE1 1DB UK

**Keywords:** Ion channels, Lipids, Membranes

## Abstract

Droplet interface bilayers (DIBs) are model membranes formed between lipid monolayer-encased water droplets in oil. Compared to conventional methods, one of the most unique properties of DIBs is that they can be connected together to generate multi-layered ‘tissue-like’ networks, however introducing communication pathways between these compartments typically relies on water-soluble pores that are unable to gate. Here, we show that network connectivity can instead be achieved using a water-insoluble membrane protein by successfully reconstituting a chemically activatable mutant of the mechanosensitive channel MscL into a network of DIBs. Moreover, we also show how the small molecule activator can diffuse through an open channel and across the neighbouring droplet to activate MscL present in an adjacent bilayer. This demonstration of membrane protein mediated bilayer communication could prove key toward developing the next generation of responsive bilayer networks capable of defining information flow inside a minimal tissue.

## Abstract (web summary)

Inserting membrane proteins into networks of droplet interface bilayers is important for building biomimetic minimal tissues. Here this is achieved using a chemically activatable mutant of the mechanosensitive channel of large conductance (MscL), where translocation of the activator through the channel defines the overall network flux.

## Introduction

Model membranes have been used for decades as biomimetic assemblies for reconstituting proteins and for studying the biophysical properties of  cell membranes^1,2^. Recently, a new class of model system has emerged that shares all of the desirable properties of conventional bilayer/black lipid membranes (BLMs), giant unilamellar vesicles and supported lipid bilayers while also supporting the ability to form extended networks of bilayers that have been demonstrated to exhibit tissue-like properties^3^. These droplet interface bilayers (DIBs) are assembled between lipid monolayer-encased water droplets in oil; lipids supplied either directly to the oil or to the aqueous phase as vesicles self-assemble at the oil–water interface and a lipid bilayer is formed when the droplets are placed into contact^4–6^. The introduction of additional droplets supports the formation of bilayer networks, which can be freely assembled in 2D or 3D^3,7–10^, while communication between compartments can be facilitated through the addition of chemical and biological motifs, such as the water-soluble pore-forming protein α-hemolysin (αHL), which self-inserts into the membrane. These capabilities have enabled the construction of ‘higher-order’ systems that exhibit collective properties, with examples including the engineering of droplet bio-batteries^7^ and current rectifiers^11^ in addition to a light-sensing network using the light-driven proton pump bacteriorhodopsin^7^. More recently, αHL-mediated connectivity between droplets has also been shown to be triggered in real time using light by exploiting a light-sensitive T7 RNA polymerase coupled to a cell-free expression system^12,13^. In view of these ground-breaking advances, it seems clear that the next milestone toward realizing the true potential of this platform technology is to demonstrate that water-insoluble membrane proteins can be successfully reconstituted into DIB networks. These far more accurately represent medically relevant targets and, critical to mediating the flow of information in synthetic systems, also offer higher functionality as a result of their ability to open and close in response to specific stimuli (αHL cannot be closed in the absence of a blocker). The major drawback is that these proteins are significantly more challenging to incorporate into membranes, owing not only to the requirement to first reconstitute them into vesicles, but also due to the difficulty of obtaining significant yields at the point of expression. Subsequently, reports of the successful reconstitution of water-insoluble membrane proteins into a two droplet (i.e. single bilayer) system have been limited to a handful of different proteins^4,14^, including ion channels and transporters expressed using cell-free extracts^15,16^; however, this has yet to be reported in networks of interconnected DIBs.

Here, we show that this can be realized to yield a new type of membrane protein-mediated communication between lipid bilayers by reconstituting a chemically activatable mutant of the mechanosensitive channel of large conductance (MscL) into networks of DIBs. MscL is an ideal candidate for functionalising DIBs as it: (a) can open to form a relatively large (3–4 nm) channel^17,18^; (b) has been extensively characterized^19,20^; (c) can be mutated to exhibit desirable characteristics, such as chemical sensitivity to methanethiosulfonate (MTS) reagents (e.g. G22C, as used in this study)^21^ and low-tension gating (e.g. V23T or G22S)^22,23^; and (iv) has been previously reconstituted in DIBs^24,25^ and bilayers formed at the interface of aqueous droplets and hydrogels (droplet hydrogel bilayers)^26^. The limitations are that MscL is not ion selective and does not tend to exhibit well-defined single-channel gating properties—instead, the channel can pass through multiple different substates before fully opening and may transition between states once opened^27^.

By inserting silver/silver chloride electrodes into the terminal droplets of DIB networks, we use electrical measurements to show that the chemical activator [2-(trimethylammonium)ethyl] methane thiosulfonate bromide (MTSET) can activate MscL, diffuse through the open channel and trigger the opening of MscL in a neighbouring bilayer. These results demonstrate membrane protein-mediated bilayer interactions inside a network of lipid bilayers and represent the next step towards unlocking greater functionality in synthetic systems by embedding water-insoluble membrane proteins that are able to sense and respond to user-engineered stimuli.

## Results

### Membrane protein purification and reconstitution into vesicles

**Fig. 1:**
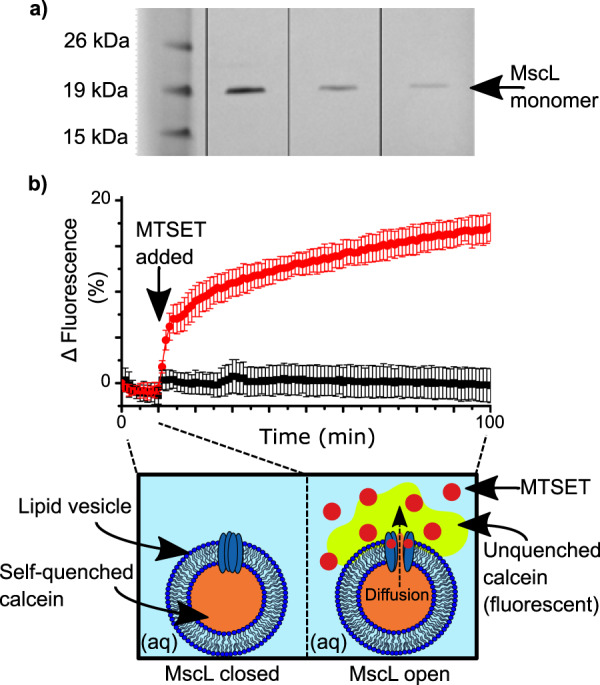
MscL purification and reconstitution in lipid vesicles. **a** SDS–PAGE gel (pre-cast NuPAGE 12% bis-tris protein gel) containing purified protein. Lane 1 contains Novex Sharp Protein Standard Ladder and lanes 2–4 contain 0.27 mg ml^−1^, 0.11 mg ml^−1^ and 0.05 mg ml^−1^ of purified protein, respectively. MscL monomer is indicated at 17 kDa. **b** Calcein release assay showing the activation of reconstituted MscL by the chemical activator MTSET. MscL is activated after 10 min by the addition of MTSET (red circles), which leads to an increase in fluorescence caused by the diffusion and subsequent dilution of quenched calcein as it diffuses out of the vesicle through the open MscL channel (red circles). No increase in fluorescence is observed when MTSET is replaced with pure buffer solution (black squares). Data were normalized to the value at time = 0 min. Errors are shown as the standard deviation of four measurements.

G22C F93W MscL was expressed and purified from *Escherichia coli* BL21 (DE3) cells carrying the kanamycin-resistant pET-28a vector using a modified protocol reported by Perozo et al.^28^. This mutant differs from the wild-type protein in two ways: (1) the G22C point mutation replaces a glycine with a cysteine in position 22, allowing it to react with MTS compounds such as MTSET to trigger channel gating; (2) the F93W point mutation replaces a phenylalanine with a tryptophan to facilitate quantification of the protein via absorbance at 280 nm. Purified protein was visualized via sodium dodecyl sulfate-polyacrylamide gel electrophoresis (SDS-PAGE) and quantified via tryptophan absorbance. A typical gel is shown in Fig. 1a. The presence of only a single band in lanes 2–4 shows that the purification was successful, while the position of the bands at ~17 kDa is in line with previous reports for the MscL monomer^29^. The contrasting intensities of the bands owes to the different amounts of protein loaded and serves as further confirmation of successful protein expression and purification. Prior to single-channel analysis, the activity of purified MscL was screened by reconstituting the protein into calcein-loaded vesicles and performing a release assay. The concept of the assay is illustrated in the schematic provided in Fig. 1b: calcein encapsulated within the vesicles is self-quenched at 50 mM and yields a negligible fluorescent signal. Upon the addition of MTSET, the MscL channels open and calcein diffuses out of the vesicles into the external aqueous medium, where it becomes unquenched to yield a strong fluorescent signal. As shown in our measurements in Fig. 1b, in the absence of MTSET, we observed no change in the fluorescence of our calcein encapsulated MscL vesicles for the first 10 min of the assay. At this point, either 1 mM MTSET in buffer (red circles) or pure buffer solution (black squares) was added to the samples. Here, a sharp and continual increase in fluorescence was observed for the vesicles exposed to MTSET, while no change in fluorescence was recorded for the control samples (*n* = 4). This leads us to conclude that the addition of MTSET triggers the opening of MscL, confirming that our protein is active. The scale of the increase in fluorescence is in-line with what has been observed in our lab previously and most likely owes to the small volume of the vesicles^25^. These results demonstrate the successful reconstitution of active MscL into lipid vesicles, which is essential for assembling MscL functionalized DIBs.

### MscL activity in DIBs

**Fig. 2:**
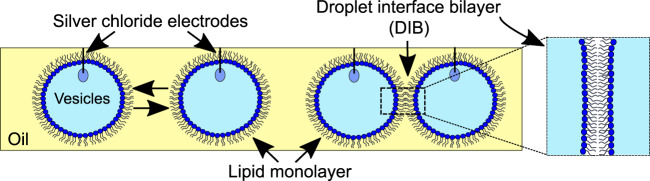
Assembly of droplet interface bilayers. Water droplets comprised of lipid vesicles are placed onto the agar-coated tips of silver/silver chloride electrodes and lowered into a well of oil. After a brief incubation period, a highly packed lipid monolayer assembles at the water–oil interface and a lipid bilayer is formed when the droplets are manipulated into contact (as highlighted in the zoom).

**Fig. 3:**
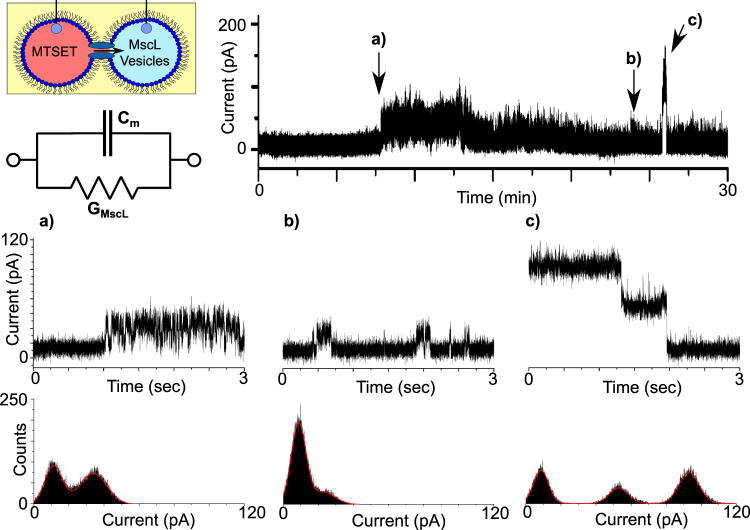
MscL activity in droplet interface bilayers. DIBs formed with reconstituted protein and MTSET give rise to discrete channel activity. A schematic of the experimental set-up is provided together with an equivalent electrical circuit, where *C*_m_ depicts the membrane capacitance and *G*_MscL_ the channel conductance. The indicated regions of a representative 30 min recording obtained at 100 mV are magnified in the 3 s segments shown in **a**–**c**. The recordings show rapid ~20 pA opening and closing events in **a**, more stable ~14 pA events in **b** and two clearly defined ~40 pA closing events in **c**. The histograms supplied with each trace reflect the different open states observed.

Prior to assembling networks of bilayers, electrical measurements of MscL (electrical set-up detailed in Supplementary Fig. 1) were first performed in DIBs comprised of two 1 μl droplets (Fig. 2). We found that we were able to reproducibly assemble DIBs with negligible leakage currents and lifetimes >30 min when both droplets consisted of extruded vesicles in buffer alone (Supplementary Fig. 2). This measurement was performed routinely prior to performing any experiments in order to identify any background noise or trace contaminants that would lead to the appearance of artefacts in our recordings or compromise the integrity of our bilayers. We observed similar results when MTSET was added to one of our droplets without MscL (Supplementary Fig. 3), confirming that MTSET alone does not destabilize or lead to channel-like activity in our membranes. In all cases, the presence of a bilayer was confirmed by measuring the bilayer capacitance (Supplementary Fig. 4). Only when reconstituted MscL was present in the second droplet did we observe activity in the membrane, appearing as discrete opening and closing, or successive opening/opening and closing/closing events (Fig. 3). While MscL channel activity could be reliably obtained, multichannel activity was often observed, and single-channel activity was difficult to decouple. A representative example of the range of different single-channel gating events we frequently observed during our 30 min recordings are highlighted in Fig. 3a–c (further examples can be found in Supplementary Fig. 5).

A region of the first burst of activity at ca. 8 min is magnified in Fig. 3a and shows rapid (ms) switching between two states lasting for several minutes. A histogram of the segment shows two clear peaks at 11.20 and 33.81 pA, corresponding to an opening of ca. 22.61 pA (0.23 nS), while built-in single-channel analysis yielded a similar value of 19.78 ± 2.31 pA (*n* = 313). In contrast, the activity highlighted in Fig. 3b shows more stable switching at ~23 min between the open and closed state albeit at a lower conductance. This is shown both in the histogram, with centrepoints at 8.73 and 22.75 pA corresponding to an opening of ca. 14.02 pA (0.14 nS), and via single-channel analysis which generates a value of 14.40 ± 2.28 pA (*n* = 495). This period of stable gating precedes a burst of relatively high conductance gating that appears at ~27 min. The extremely well-defined transition states that conclude this burst of activity are highlighted in Fig. 3c. The histogram shown as part of Fig. 3 highlights three peaks at 8.59, 52.97, and 93.02 pA, indicating a drop of 40.05  pA (0.40 nS), followed by 44.38 pA (0.44 nS) as the signal returns to baseline at 8.59 pA. It is unclear whether this type of activity is the result of the transition between two different open states, or whether this depicts a scenario where two channels in the same open state are closing in succession. The magnitude of these openings (i.e. ~0.40 nS) are similar to what have been reported previously for this mutant^21,30,31^, which shows a preference for the same subconducting state when gated by MTSET in the absence of tension^31^, supporting the latter hypothesis. However, as MscL can exhibit multiple sub-conductance states, the former hypothesis cannot be dismissed^19^. In any case, it is clear that our MscL is functional in DIBs, and this platform is suitable for measuring MscL channel activity.

### MscL activity in DIB networks

**Fig. 4:**
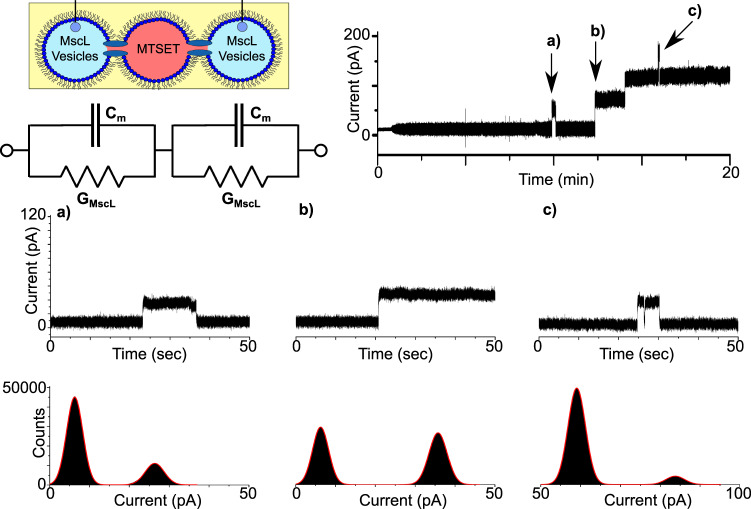
MscL activity in networks of droplet interface bilayers. Three droplet DIB networks were formed with reconstituted protein in the first and last droplet and MTSET in the middle droplet. A representative 20 min bilayer current recording shows multiple gating events including successive openings. A schematic of the experimental set-up is provided together with an equivalent electrical circuit, which highlights that the droplets are connected in series, leading to a reduction in the total capacitance and an increase in the effective resistance, resulting in a reduction in the bilayer current. The indicated regions of the trace are magnified in the 50 s segments shown in **a**–**c**. The recording shows an ~20 pA opening and closing event that lasts ca. 12 s in **a**, an ~30 pA opening that leads to multiple opening events in **b** and an ~20 pA third-level burst of activity **c**. The histograms supplied with each trace reflect the different open states recorded.

To show that MscL could be successfully reconstituted into a network of DIBs, we introduced a third 1 µl droplet into our system and positioned it between the two droplets attached to the Ag/AgCl electrodes. As a control, the droplets were initially supplied with vesicles in the absence of protein. In this configuration, we observed no activity in our measurements, but noted that the peak-to-peak noise of the baseline was significantly lower than a two-droplet system (Supplementary Fig. 6), most likely due to the reduction of the bilayer capacitance owing to the two bilayers being connected in series (Supplementary Fig. 4). We subsequently supplied each terminal droplet with reconstituted protein and dissolved MTSET into the middle droplet (Fig. 4). With the MTSET directly accessible to MscL reconstituted in each bilayer, we observed distinct opening/closing and opening/opening events in our measurements that lasted for up to several minutes, suggesting that MscL was simultaneously active in both membranes. The indicated regions of a representative 20 min recording (*n* = 3, Supplementary Fig. 7) are expanded in Fig. 4a–c to display 50 s zooms of the trace. Figure 4a shows a ~12-s-long opening event at ~10 min, which can be clearly observed as two well-defined peaks at 6.27 and 26.34 pA in the corresponding histogram. This equates to a 20.07 pA (0.2 nS) opening, which is similar to the value of 22.29 ± 1.41 pA (*n* = 11,842) generated by single-channel analysis. In contrast, the data in Fig. 4b show the first of two successive openings at ~13 min, which appears to be slightly more intense compared to the zoom in Fig. 4a. The associated histogram reveals peaks at 6.25 and 35.75 pA, which corresponds to an opening of 29.5 pA (0.30 nS) and is in close agreement with the value of 29.88 ± 1.31 pA (*n* = 2759) obtained from single-channel analysis of the trace. The zoomed region in Fig. 4c shows a 5-s-long third level opening at ~16 min, which also exhibits a brief closing/opening event, indicative of channel gating. The histogram shows peaks at 59.05 and 83.77 pA, resulting in an opening of 24.72 pA (0.25 nS), which was similar to the value of 21.06 ± 1.31 pA (*n* = 17) calculated via single-channel analysis.

As the droplets containing reconstituted MscL are connected in series, it is expected that the total resistance of the circuit should reflect the sum of the individual resistor values as highlighted in the equivalent circuit diagram shown in Fig. 4; assuming that both channels are in the same conductance state, the measured current should be approximately half of what was measured for a single DIB^32^. This appears to be in line with our findings with the ~20 pA values obtained in Fig. 4a, c appearing almost exactly half of the ~40 pA openings found in Fig. 3c, suggesting that in each example MscL is open in a subconducting state in both bilayers. While the origins of the ~29 pA openings recorded in Fig. 4b are less clear, it likely they originate from active MscL in different conducting states simultaneously. Our results confirm that MscL has been reconstituted into a network of interconnected DIBs, which to our knowledge is the first example of a functional water-insoluble membrane protein being successfully implanted into a bilayer network.

### MscL-mediated bilayer communication in DIB networks

**Fig. 5:**
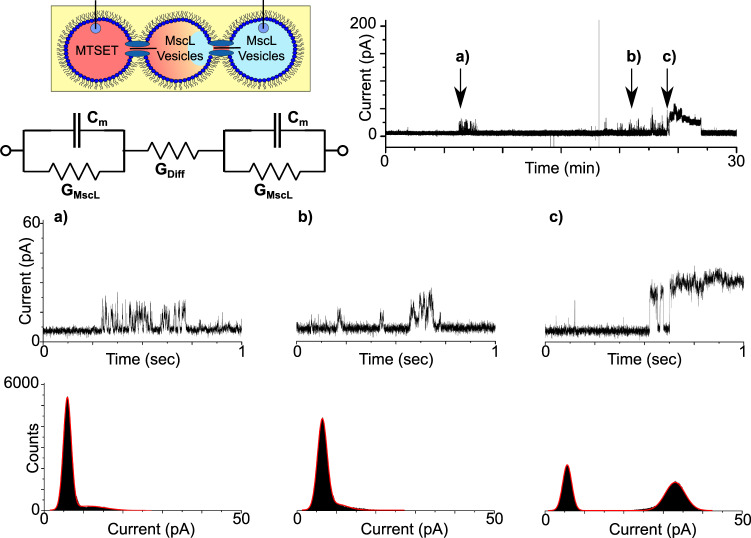
MscL-mediated bilayer communication in DIB networks. Three droplet DIB networks were formed with MTSET in the first droplet and MscL vesicles in the second and third as highlighted in the schematic. As lipid bilayers are impermeable to the free diffusion of MTSET, the only route for network activation is for passive diffusion through the open channel and across the adjacent droplet to activate MscL in the second membrane. The time taken for this to take place is reflected in the equivalent circuit through the addition of an extra resistor labelled G_Diff_ (this is not intended to indicate a further reduction in the bilayer current). A representative 30-min bilayer current recording shows a short burst of activity at ~6 min and more significant activity from 20 min onward. The indicated regions of the trace are magnified in the 1 s segments shown in **a**–**c**. The zooms shows a brief ~7 pA burst of activity in **a**, a similar ~7 pA burst of activity in **b** and two brief ~24 pA opening and closing events in **c** that lead to the channel being held open. The histograms supplied with each trace reflect the different open states observed in the recordings and emphasize the low number of open events in **a**, **b**.

To further demonstrate that MscL could be successfully reconstituted into DIB networks, we rotated the positions of the first two droplets in Fig. 4 such that the droplet containing MTSET was placed first. In this configuration, activation of the network can only be achieved by the MTSET activating MscL in the first bilayer and diffusing through the open channel and across the neighbouring droplet to activate MscL embedded within the second bilayer. In this sense, the state of MscL in the second bilayer is entirely dependent on the state of MscL in the first bilayer, establishing a form of MscL-mediated bilayer communication across the network (Fig. 5).

Under these conditions, we observed noticeably fewer events in our measurements compared to our previous results. A representative 30-min trace (*n* = 3, Supplementary Fig. 8) is supplied in Fig. 5 together with 1 s zooms and histograms. Figure 5a shows a short burst of rapid (ms) opening and closing events at ~6 min. These events appear on the histogram as a baseline peak at 5.88 pA and an almost indistinguishable second peak at 11.10 pA, corresponding to openings of 5.22 pA (0.05 nS). Single-channel analysis generated a value of 7.39 ± 1.80 pA (*n* = 164). Fewer events of similar magnitude were also recorded at ~21 min in Fig. 5b, with histograms revealing peaks at 6.41 and 9.82 pA, yielding openings of 3.41 pA (0.03 nS) and corresponding to a value of 6.09 ± 1.41 pA (*n* = 195) from single-channel analysis. More pronounced events are shown in Fig. 5b at ~24 min with two brief opening/closing events recorded before the channel was held open for several minutes. The associated histogram reveals peaks at 5.63 and 32.99 pA resulting in an opening of 27.36 pA (0.27 nS), slightly higher than the value of = 23.62 ± 1.04 pA (*n* = 1072) generated via single-channel analysis. We observed no activity when the droplets containing MscL vesicles were substituted for vesicles in buffer (*n* = 3, Supplementary Fig. 9).

As the droplets in Fig. 5 are connected in series as in Fig. 4, it is similarly expected that the channel current should be approximately half of what was measured for MscL reconstituted inside a single DIB, assuming that MscL in both membranes is in the same open state. This appears to be consistent in our data for both Fig. 5a, b, with the ~7 pA openings representing half of the ~14 pA events recorded in Fig. 3b. Additionally, the ~24 pA opening measured in Fig. 5c matches well with results from Fig. 4c, and is attributed to subconductance opening of MscL channels in each bilayer (due to current magnitudes half of the ~40 pA opening observed in Fig. 3c) or alternatively a combination of multiple conductance states across the set of bilayers.

The feasibility of MTSET (approximate diameter of 0.58 nm^33^) diffusing through the open channel and across the neighbouring droplet to activate MscL in the second bilayer is also supported by MscL possessing a relatively large, unselective pore with a diameter of ~3–4 nm in the open state^17,18,34^. MTSET diffusion through the pore is therefore expected, especially as much larger fluorescent molecules^25,35^, such as peptides and proteins^36^, have previously been shown to translocate through the open MscL channel, even when primarily gating in the subconductance states triggered by MTSET (molecular weight cut-off ~6.6 kDa)^31^. Using a simple approximation from Fick’s First Law and the Stokes–Einstein equation to calculate the diffusion coefficient of MTSET, we can estimate the concentration of MTSET in the second droplet over time assuming one sub-gating MscL channel (*d* = 1.5 nm)^19^. This shows that in the time frame of the experiment, an MTSET equilibrium would not be reached and that the amount of MTSET in the second droplet increases slowly, reaching beyond the low MTSET low concentration required to open MscL of only five molecules required for the five subunits of the MscL pentamer (Supplementary Fig. 10). To account for this delay, an additional resistor is also added to the equivalent circuit in Fig. 5.

It is of note that we observe some activity ahead of the main MscL activity in our measurements, which is expected given that the first droplet is supplied with excess MTSET and, in principle, <5 molecules are required to activate the channel. However, it is clear from Fig. 5a, b that channel activity is brief, less clearly defined and less frequent compared to the rest of our recordings, which is likely due to the lack of significant MTSET concentrations in the middle droplet for the first 22 min of the recording (also observed in Supplementary Fig. 8). This may not account for rapid opening/closing events as individual MTSET molecules bind and form a covalent S–S linkage with cysteine in each MscL subunit, but the distorted opening/closing events observed in Fig. 5 can arise due to transient behaviour of the two MscL channels across two bilayers^32^.

These factors support our conclusion that MscL is active in DIB networks, indicating that the opening of MscL by MTSET present in the first bilayer did indeed trigger the gating of MscL in the second, especially as it is known that MTSET cannot passively diffuse across lipid bilayers^37,38^. Our findings demonstrate membrane protein-mediated bilayer communication in a synthetic system and could pave the way for developing more biomimetic signalling and communication pathways in DIBs.

## Discussion

Here we have demonstrated the successful integration of water-insoluble protein channels into DIB networks, confirming the activity of the MscL protein in a variety of droplet configurations. After confirming channel activity in a single DIB (Fig. 3) and across a three-droplet minimal network (Fig. 4), we confirm that soluble channel activators can be used to propagate a chemical signal through the network upon sequential channel activation (Fig. 5). Our findings present a significant advance towards the development of artificial tissues that more closely replicate the cell–cell interactions found in natural biological systems. This substantial level of biomimicry, both in terms of the functionalization of our lipid bilayers and the interplay between them, could have a profound impact on the next generation of smart drug delivery systems, screening models and compartmentalized bioreactors designed to respond to user-defined stimuli. While we employ a chemical stimuli coupled to MscL reconstituted in bilayer networks, this could also be achieved with the same channel via mechanical stimulation^24^, protein–protein interactions^39^ or in combination with other channels or transporters^16^ together with the appropriate activator, creating pathways of chemical flux defined by extended DIB coupling. This flexibility coupled with the freedom to assemble DIB networks of almost infinite size and geometry, with ever-increasing biomimetic composition^40^ and the ability to incorporate light-triggered enzymatic reactions^41^ and cell-free protein expression into these systems could present paradigm shifting fidelity with almost endless possibilities for generating responsive, next-generation artificial tissues.

## Methods

### Lipid preparation

1,2-Dioleoyl-*sn*-glycero-3-phosphocholine (DOPC) and 1,2-dioleoyl-*sn*-glycero-3-phosphoglycerol (DOPG) were obtained from Avanti Polar Lipids Inc. (USA). SM2 Bio-Beads (mesh size 20–50) were purchased from Bio-Rad. MTSET was purchased from Biotium. All other compounds were purchased from Sigma-Aldrich (UK).

### MscL expression and purification

*Escherichia coli* BL21 (DE3) cells carrying the kanamycin-resistant pET28a vector were expressed using a modified protocol of Perozo et al.^28^. MscL was purified using an adapted protocol reported by Charalambous et al.^39^. *Escherichia coli* BL21 (DE3) cells carrying the pET28a (Novagen) vector with the MscL gene (with G22C and F93W mutations) were grown overnight in Luria Broth (LB) (30 µg ml^−1^ kanamycin) at 37 °C and 200 r.p.m. The overnight culture was reseeded 1:100 into 1 L fresh LB (30 µg ml^−1^ kanamycin) and incubated at 37 °C and 200 r.p.m. to ~maximum of the exponential phase (OD_600_ = 1). Protein production was induced for 45 min in the presence of isopropyl β-d-thiogalactopyranoside (0.5 mM). Cells were collected by centrifugation at 16,000 × *g* (45 min, 4 °C) and resuspended in 50 ml of phosphate-buffered saline and 0.1 mM phenylmethanesulfonylfluoride (PMSF) solution. Cells were lyzed by two passes through a cell disruptor (Constant Systems Cell Disruptor Model T5) at 25 KPSI. Membrane fractions were isolated through centrifugation (100,000 × *g*, 1 h, 4 °C) and solubilized in solubilization buffer (20 mM HEPES pH 7.2, 100 mM KCl, 2% (w/v) dodecyl-β-maltopyranoside (DDM), EDTA-free protease inhibitor) overnight with rotation. Insoluble material was removed by centrifugation at 100,000 × *g* for 30 min at 4 °C. The supernatant was diluted 2-fold in 20 mM HEPES pH 7.2, 100 mM KCl and DDM-solubilized protein batchbbound to 0.67 ml TALON cobalt metal affinity resin per litre of LB for 90 min at 4 °C with rotation. The resin was centrifuged at 1000 × *g* until a pellet was formed (5–10 min) and the supernatant removed.

Non-specifically bound protein was removed by washing the resin with 40 ml wash buffer (20 mM HEPES pH 7.2, 100 mM KCl, 0.13% DDM, 0.1 mM PMSF and 6 mM imidazole) and incubating the suspension for 10 min at 4 °C with rotation. The resuspended resin was pelleted again via centrifugation (1000 × *g*, 5–10 min) and the supernatant removed. The pellet was resuspended in 10 ml of wash buffer, transferred to a gravity-flow column and the resin was allowed to settle out of suspension. MscL was eluted in 2.5 ml elution buffer (20 mM HEPES pH 7.2, 100 mM KCl, 0.13% DDM, 0.1 mM PMSF, 150 mM imidazole) per litre of LB and prepared to a final concentration of 0.43 mM using an Amicon Ultra 100,000 MWCO centrifugal concentrator (Millipore). Imidazole was removed using a PD-10 desalting column (GE Healthcare) and the protein was further concentrated using an Amicon Ultra 100,100 MWCO centrifugal concentrator. Purified MscL was quantified using a NanoDrop 2000 UV-Vis Spectrophotometer (Thermo Scientific) by measuring the absorbance at 280 nm. Samples were snap frozen in liquid nitrogen and stored at −80 °C until reconstitution. For consistency, all experiments contained MscL from one expression and purification.

### MscL reconstitution

Five milligrams of 95:5 DOPC:DOPG (mol:mol) were dissolved in a minimum of chloroform, evaporated under nitrogen and dried overnight in a lyophilizer. Dried films were then hydrated with 20 mM HEPES, 100 mM KCl, 40 mM *n*-octyl-β-d-glucopyranoside pH 7.4 buffer. For calcein release experiments, 50 mM calcein was also present in the suspension buffer. Four freeze–thaw cycles were applied before extruding the suspension 11 times through a 100-nm filter to yield a detergent-saturated vesicle population. MscL was added at various lipid:MscL ratios, and the detergent was removed by the addition of 100 mg of SM2 Bio-Beads to yield vesicles at 4 °C. Vesicles were incubated with Bio-Beads for an hour before removing the beads and repeating the process twice more. After 3 h of incubation, Bio-Beads were removed and stored at 4 °C until use. As a control, in the absence of MscL, an equivalent amount of DDM was added to the detergent-saturated vesicles, and the same detergent removal process was followed.

For the calcein release assay, after Bio-Bead removal the sample was diluted 140-fold in 20 mM HEPES, 100 mM KCl buffer pH 7.4. Ultracentrifugation (130,000 × *g*, 4 °C, 1 h) yielded a vesicle pellet, which was resuspended in sucrose buffer (500 mM sucrose, 20 mM HEPES, 100 mM KCl, pH 7.4) as present before dilution. The vesicles were then stored at 4 °C until further use. For single-channel electrophysiology, 4.5 µg protein was supplied to 10 mg ml^−1^ total lipid.

### DIB formation

Glass slides were spin coated with polydimethylsiloxane (PDMS) (Sylgard 184, Dow Corning) at a ratio of 10:1 (base to curing agent) and cured at 90 °C for 10 min. Wells were laser cut from polymethyl methacrylate and fixed to the polydimethylsiloxane-coated slide using double-sided adhesive prior to filling with hexadecane. 5% agar (w/v) in deionized water was dispensed onto Ag/AgCl electrodes and washed extensively with a buffer (100 mM KCl, 20 mM HEPES, pH 7.4). 1 μl of the appropriate proteoliposome dispersion was placed onto the agar-coated tips of the electrodes and lowered into the hexadecane wells via micromanipulators (Thorlabs). The droplets were left for 5–10 min to promote the assembly of a highly packed lipid monolayer before being placed into contact. For the assembly of a three-droplet network, the additional droplet was dispensed into the well and sandwiched between the two existing droplets. At the end of the measurement, the droplets were separated and dragged to the edge of the well where the electrode was withdrawn. The electrodes were thoroughly washed with buffer and the agar regularly replaced in between experiments.

### Electrical measurements and analysis

Electrophysiology was performed using an Axopatch 200B amplifier and an Axon Digidata 1440A digitizer in combination with a CV-203BU headstage (Molecular Devices). Current measurements were obtained using a holding potential of 100 mV, a 5 kHz low-pass 8-pole Bessel filter, and a sampling rate of 50 kHz. For presentation purposes, the current traces were then digitally filtered using a 1 kHz low-pass filter and reduced to a sampling interval of 120 µs. Electrical measurements were analysed using the built-in functions within ClampFit (Molecular Devices). The peaks identified on each histogram were used to position the appropriate cursor for single-channel analysis.

## Supplementary information

Supplementary Information

## Data Availability

The datasets generated during and/or analysed during the current study are available from the corresponding author upon reasonable request.

## References

[1] Siontorou C, Nikoleli G-P, Nikolelis D, Karapetis S (2017). Artificial lipid membranes: past, present, and future. Membranes.

[2] Simons K, Vaz WL (2004). Model systems, lipid rafts, and cell membranes. Annu. Rev. Biophys. Biomol. Struct..

[3] Villar G, Graham AD, Bayley H (2013). A tissue-like printed material. Science.

[4] Bayley H (2008). Droplet interface bilayers. Mol. Biosyst..

[5] Funakoshi K, Suzuki H, Takeuchi S (2006). Lipid bilayer formation by contacting monolayers in a microfluidic device for membrane protein analysis. Anal. Chem..

[6] Osaki T, Takeuchi S (2017). Artificial cell membrane systems for biosensing applications. Anal. Chem..

[7] Holden MA, Needham D, Bayley H (2007). Functional bionetworks from nanoliter water droplets. J. Am. Chem. Soc..

[8] Wauer T (2014). Construction and manipulation of functional three-dimensional droplet networks. ACS Nano.

[9] Friddin MS (2016). Optically assembled droplet interface bilayer (OptiDIB) networks from cell-sized microdroplets. Soft Matter.

[10] Booth MJ, Restrepo Schild V, Downs FG, Bayley H (2017). Functional aqueous droplet networks. Mol. Biosyst..

[11] Maglia G (2009). Droplet networks with incorporated protein diodes show collective properties. Nat. Nanotechnol..

[12] Booth, M. J., Schild, V. R., Graham, A. D., Olof, S. N. & Bayley, H. Light-activated communication in synthetic tissues. *Sci. Adv.* **2**, 10.1126/sciadv.1600056 (2016). 10.1126/sciadv.1600056 PMC4820383 27051884

[13] Booth MJ, Restrepo Schild V, Box SJ, Bayley H (2017). Light-patterning of synthetic tissues with single droplet resolution. Sci. Rep..

[14] Aghdaei S, Sandison ME, Zagnoni M, Green NG, Morgan H (2008). Formation of artificial lipid bilayers using droplet dielectrophoresis. Lab Chip.

[15] Friddin MS (2013). Single-channel electrophysiology of cell-free expressed ion channels by direct incorporation in lipid bilayers. Analyst.

[16] Findlay, H. E., Harris, N. J. & Booth, P. J. In vitro synthesis of a major facilitator transporter for specific active transport across droplet interface bilayers. *Scientific Rep.* **6**, 39349 (2016). 10.1038/srep39349 PMC5172200 27996025

[17] Perozo E, Cortes DM, Sompornpisut P, Kloda A, Martinac B (2002). Open channel structure of MscL and the gating mechanism of mechanosensitive channels. Nature.

[18] Cruickshank CC, Minchin RF, Le Dain AC, Martinac B (1997). Estimation of the pore size of the large-conductance mechanosensitive ion channel of *Escherichia coli*. Biophys. J..

[19] Sukharev SI, Sigurdson WJ, Kung C, Sachs F (1999). Energetic and spatial parameters for gating of the bacterial large conductance mechanosensitive channel, MscL. J. Gen. Physiol..

[20] Blount P, Sukharev SI, Schroeder MJ, Nagle SK, Kung C (1996). Single residue substitutions that change the gating properties of a mechanosensitive channel in *Escherichia coli*. Proc. Natl Acad. Sci. USA.

[21] Yoshimura K, Batiza A, Kung C (2001). Chemically charging the pore constriction opens the mechanosensitive channel MscL. Biophys. J..

[22] Anishkin A, Chiang CS, Sukharev S (2005). Gain-of-function mutations reveal expanded intermediate states and a sequential action of two gates in MscL. J. Gen. Physiol..

[23] Yoshimura K, Batiza A, Schroeder M, Blount P, Kung C (1999). Hydrophilicity of a single residue within MscL correlates with increased channel mechanosensitivity. Biophys. J..

[24] Najem JS (2015). Activation of bacterial channel MscL in mechanically stimulated droplet interface bilayers. Sci. Rep..

[25] Barriga HM (2014). Droplet interface bilayer reconstitution and activity measurement of the mechanosensitive channel of large conductance from *Escherichia coli*. J. R. Soc. Interface.

[26] Rosholm KR (2017). Activation of the mechanosensitive ion channel MscL by mechanical stimulation of supported droplet-hydrogel bilayers. Sci. Rep..

[27] Sukharev S, Betanzos M, Chiang C-S, Guy HR (2001). The gating mechanism of the large mechanosensitive channel MscL. Nature.

[28] Perozo E, Kloda A, Cortes DM, Martinac B (2001). Site-directed spin-labeling analysis of reconstituted MscL in the closed state. J. Gen. Physiol..

[29] David MM, Heather EF, Oscar C, Richard HT, Paula JB (2016). Light-activated control of protein channel assembly mediated by membrane mechanics. Nanotechnology.

[30] Price CE, Kocer A, Kol S, van der Berg JP, Driessen AJM (2011). In vitro synthesis and oligomerization of the mechanosensitive channel of large conductance, MscL, into a functional ion channel. FEBS Lett..

[31] Mika JT, Birkner JP, Poolman B, Koçer A (2013). On the role of individual subunits in MscL gating: “All for one, one for all?”. FASEB J..

[32] Hwang WL, Holden MA, White S, Bayley H (2007). Electrical behavior of droplet interface bilayer networks: experimental analysis and modeling. J. Am. Chem. Soc..

[33] Oelstrom K, Goldschen-Ohm MP, Holmgren M, Chanda B (2014). Evolutionarily conserved intracellular gate of voltage-dependent sodium channels. Nat. Commun..

[34] Wang Y (2014). Single molecule FRET reveals pore size and opening mechanism of a mechano-sensitive ion channel. eLife.

[35] Powl AM, East JM, Lee AG (2008). Anionic phospholipids affect the rate and extent of flux through the mechanosensitive channel of large conductance MscL. Biochemistry.

[36] van den Bogaart G, Krasnikov V, Poolman B (2007). Dual-color fluorescence-burst analysis to probe protein efflux through the mechanosensitive channel MscL. Biophys. J..

[37] Holmgren M, Liu Y, Xu Y, Yellen G (1996). On the use of thiol-modifying agents to determine channel topology. Neuropharmacology.

[38] Park JS, Hughes SJ, Cunningham FKM, Hammond JR (2011). Identification of cysteines involved in the effects of methanethiosulfonate reagents on human equilibrative nucleoside transporter 1. Mol. Pharmacol..

[39] Charalambous K (2012). Engineering de novo membrane-mediated protein–protein communication networks. J. Am. Chem. Soc..

[40] Barlow, N. E. et al. Engineering plant membranes using droplet interface bilayers. *Biomicrofluidics* **11**, 10.1063/1.4979045 (2017). 10.1063/1.4979045 PMC5367087 28396711

[41] Hindley JW (2018). Light-triggered enzymatic reactions in nested vesicle reactors. Nat. Commun..

