# Supplementary material for: Membrane protein mediated bilayer communication in networks of droplet interface bilayers
Source: Commun Chem. 2020 Jun 12;3:77. doi: 10.1038/s42004-020-0322-1 (PMC7610947; doi:10.1038/s42004-020-0322-1)
Supplement: Supplementary file 1 — Supplementary Information [file 42004_2020_322_MOESM1_ESM.pdf]

Supplementary Information for:

Membrane protein mediated bilayer communication in networks of droplet interface bilayers

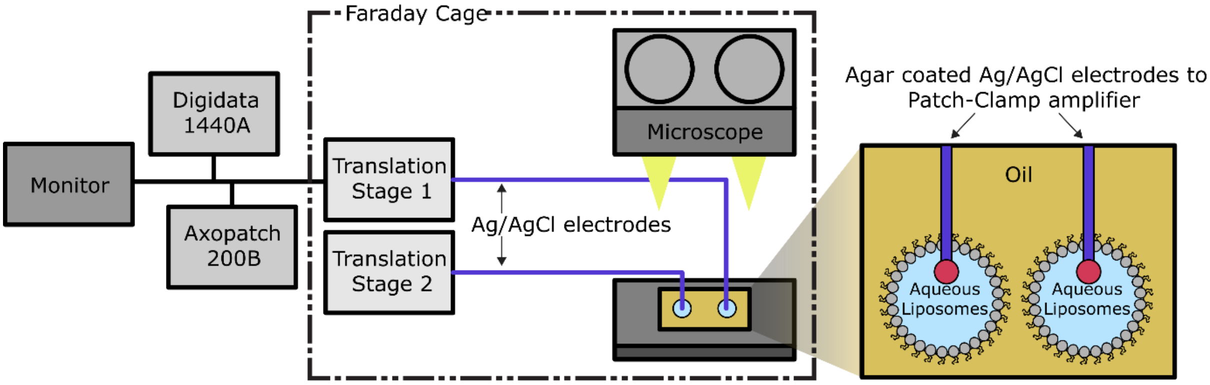

Supplementary Figure 1. Diagram of geometry of electrophysiological setup. A schematic representation of the setup for performing electrophysiological DIB measurements. The translation stage and microscope are positioned inside the faraday cage, positioned on top of the PDMS wells. Ag/AgCl electrodes are attached to the translation stage and connected to the head stage via gold-plated wires. A shielded wire connected the head stage to the amplifier, and both the amplifier and digitiser are connected to the computer by USB.

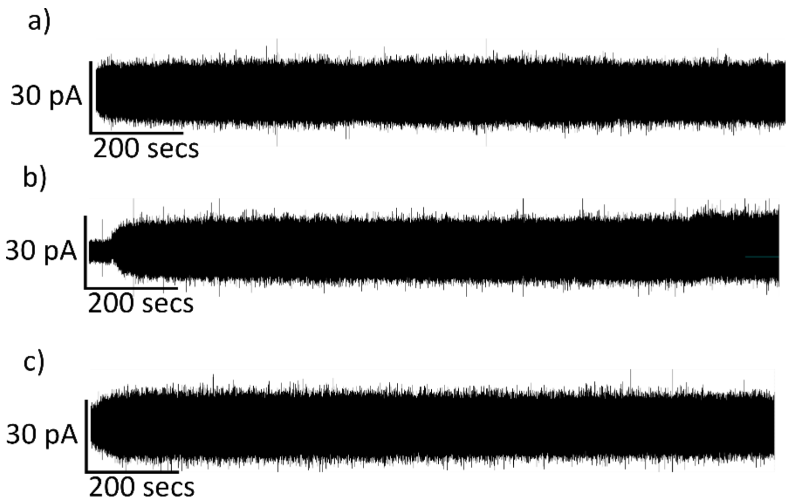

Supplementary Figure 2. MscL inclusive control data. Electrophysiological recording of a two-droplet DIB with MscL reconstitution but without the presence of MTSET in either droplet. No MscL activity is measured in the time frame of the assay, although there is a noted increase in the amount of noise. The increase in background current is due to the increase in capacitance as the DIB forms. a), b) & c) represent n=3 for these control experiments.

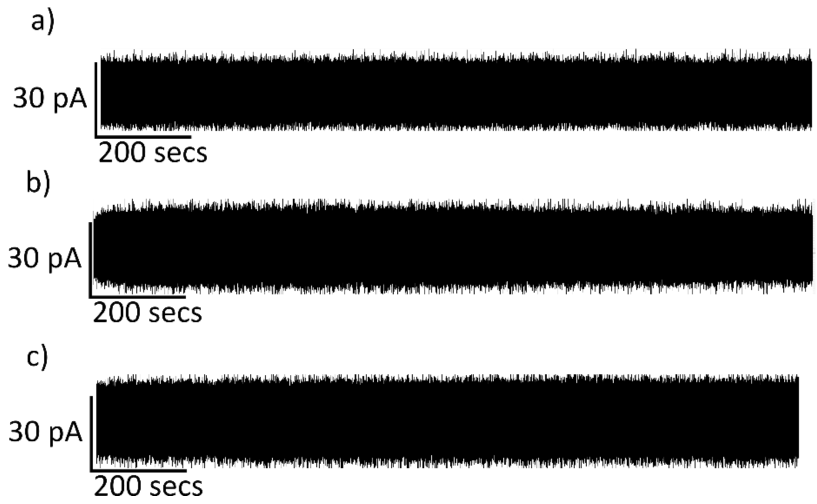

Supplementary Figure 3. MTSET inclusive control data. Electrophysiological recording of a two-droplet DIB without MscL reconstitution but with MTSET present in one droplet. No poration occurs in the membrane due to the presence of MTSET alone. a), b) & c) represent n=3 for these control experiments.

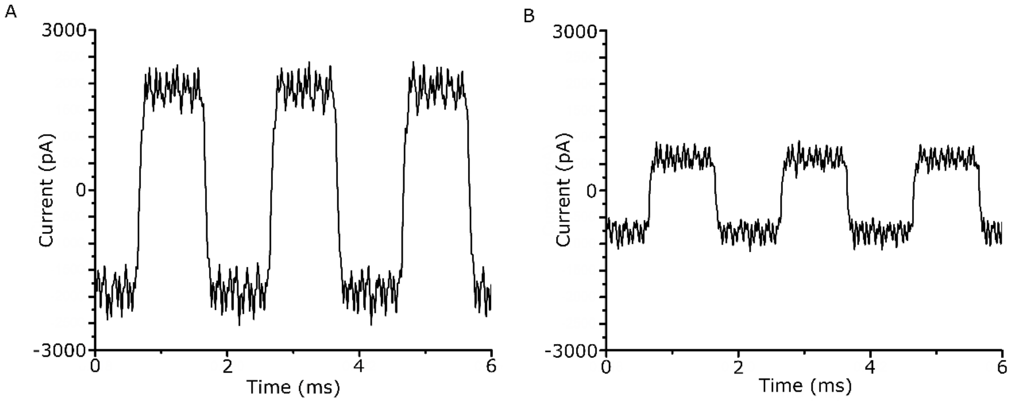

Supplementary Figure 4. Bilayer capacitance measurements of two and three droplet networks. By applying a 500 Hz triangle waveform linear voltage ramp with 1 mV peak-to-peak the bilayer current can be interpreted as bilayer capacitance as dV/dT = 1 and I = dV/dT x C. A) For a DIB assembled between two droplets we found a typical bilayer capacitance of 2 nF. B) For a three droplet DIB network, we found a typical bilayer capacitance of 750 pF. In the absence of a bilayer (i.e. when the droplets were separated) we observed a negligible background capacitance of ~20 pF, similar to what has been reported previously.[2]

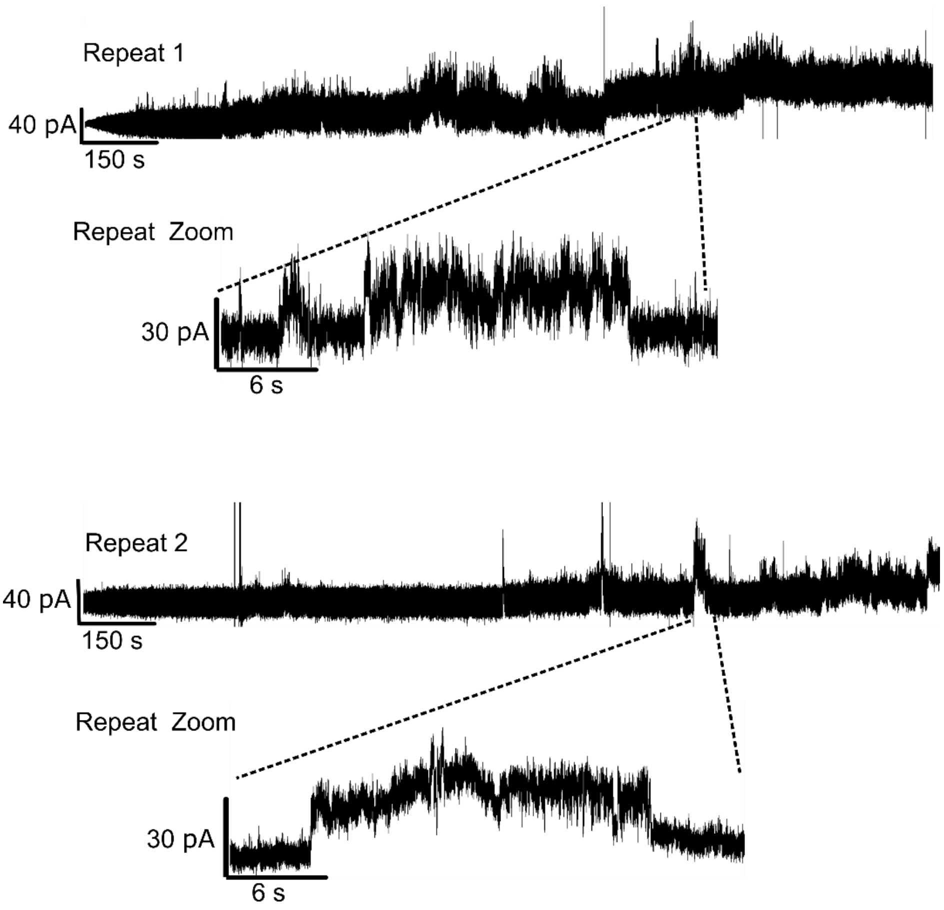

Supplementary Figure 5. Further examples of gating events. Further examples of two droplet MscL activity with MTSET present in one droplet. The region are representative of a 30 min recording obtained at 100 mV and activity is are magnified in the segments shown. The repeats show MscL opening events of approximately 30-40pA.

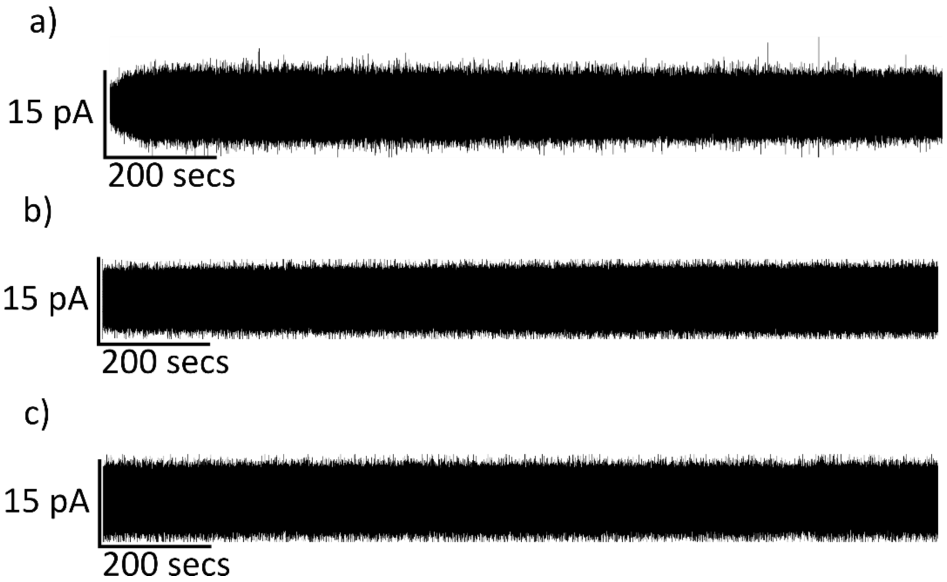

Supplementary Figure 6. MTSET inclusive 3 droplet control data with MTSET in central droplet. Electrophysiological recording of a three-droplet DIB without MscL reconstitution but with MTSET present in the middle droplet. No poration occurs in the membrane due to the presence of MTSET alone. a), b) & c) represent n=3 for these control experiments.

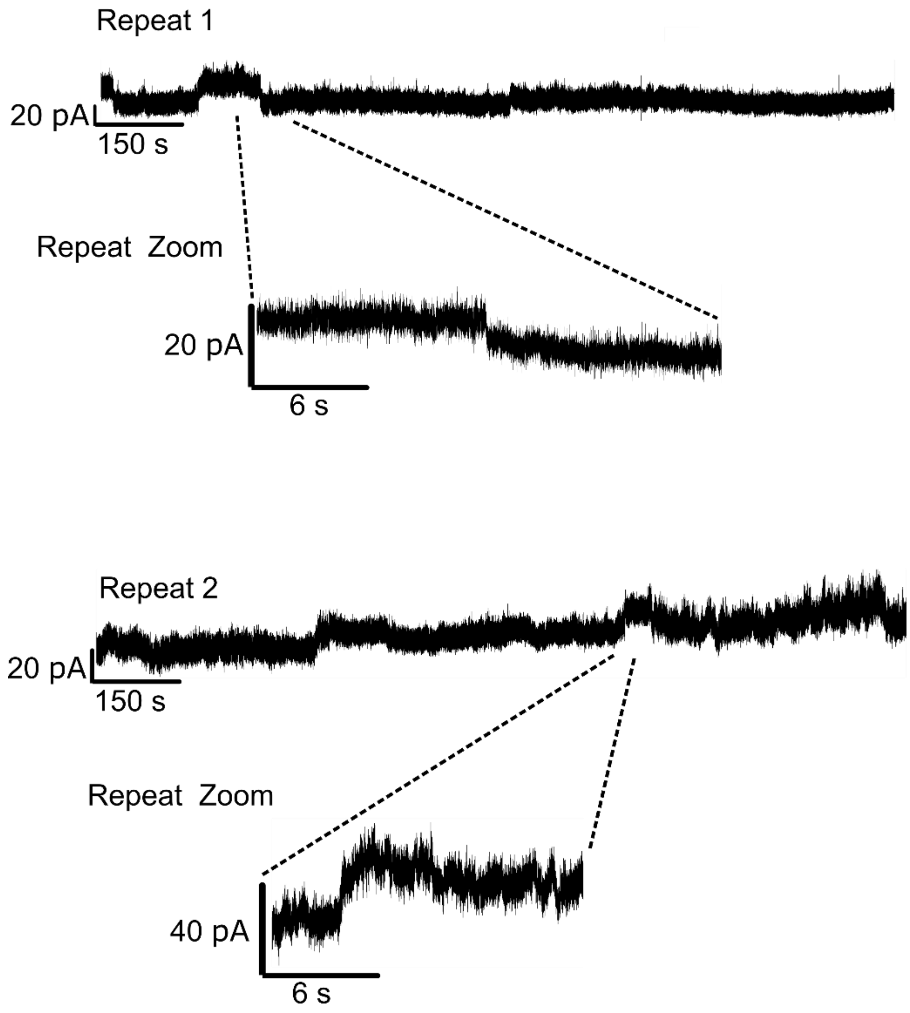

Supplementary Figure 7. Additional 3 droplet results with MTSET in first droplet. Further examples of 3 droplet MscL activity with MTSET inclusive in the second droplet. The regions are representative of a 20 min recording obtained at 100 mV and activity is are magnified in the segments shown. The repeats show MscL opening events of approximately 20pA.

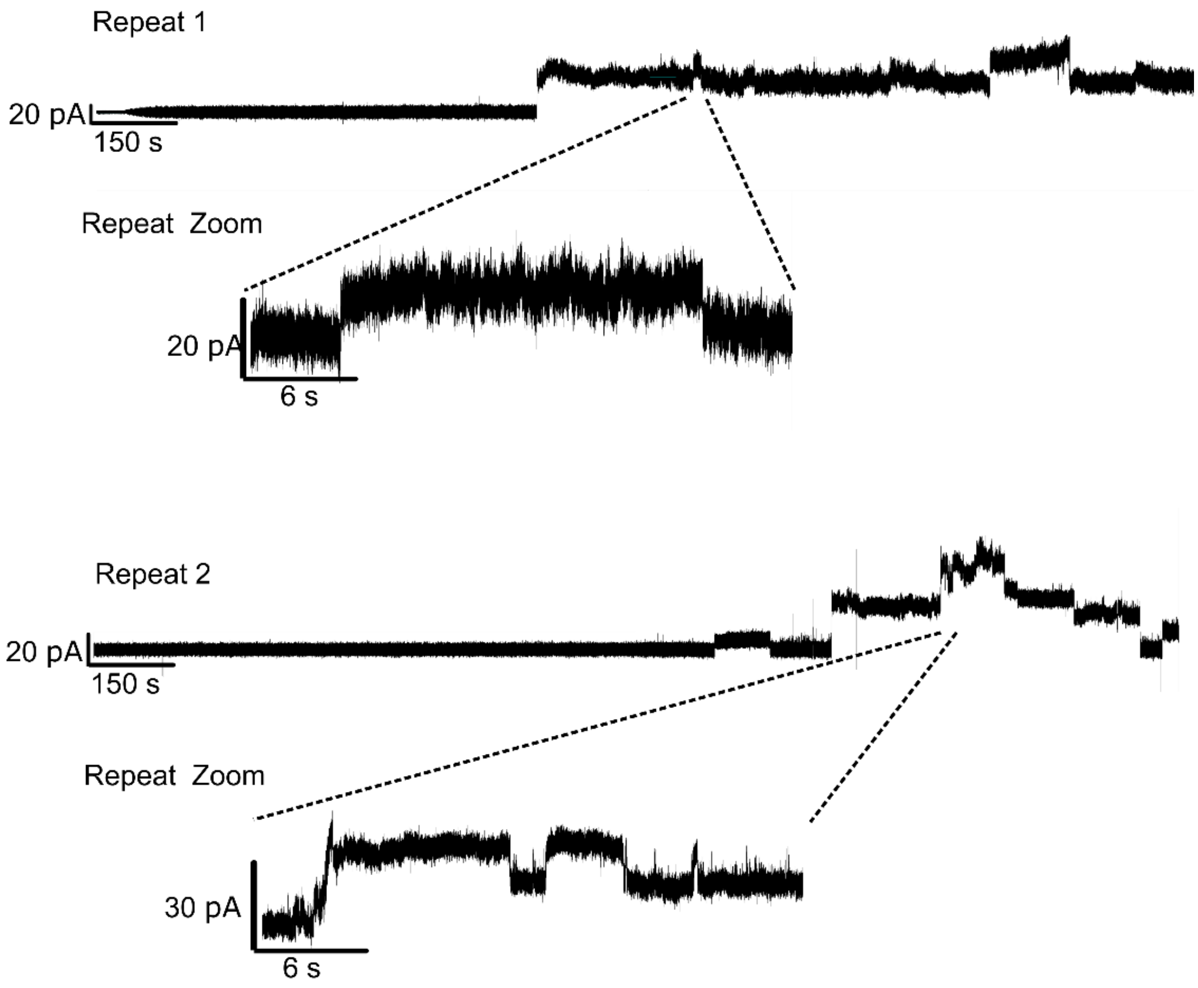

Supplementary Figure 8. Additional 3 droplet results with MTSET in first droplet. Further examples of 3 droplet MscL activity with MTSET inclusive in the first droplet. The regions are representative of a 30 min recording obtained at 100 mV and activity is are magnified in the segments shown. The repeats show MscL opening events of approximately 20pA. The delay before the first MscL activity differs between the three results and can be attributed to the diffusion time of MTSET from the first droplet, through the MscL pore, to the second droplet.

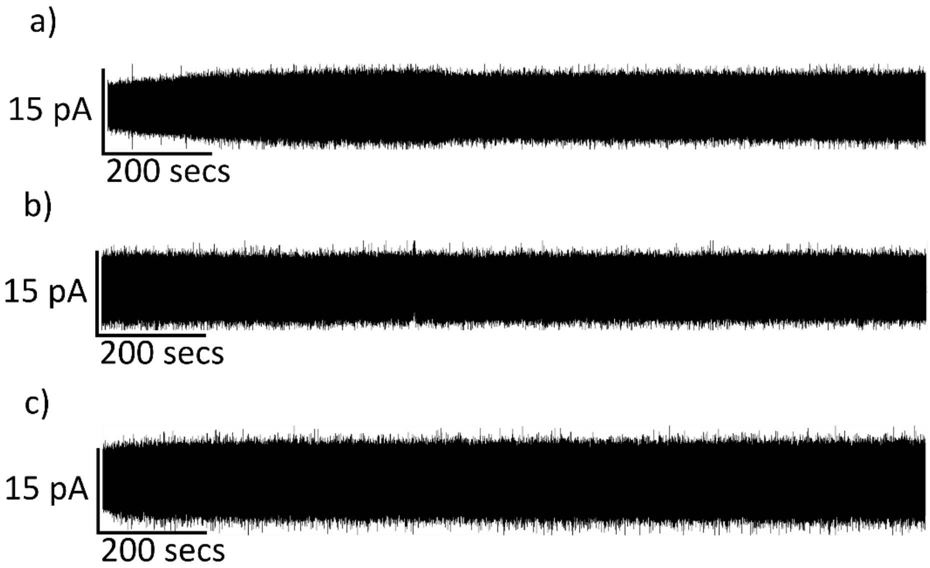

Supplementary Figure 9. MTSET inclusive 3 droplet control data with MTSET in first droplet. Electrophysiological recording of a three-droplet DIB without MscL reconstitution but with MTSET present in the first droplet. No poration occurs in the membrane due to the presence of MTSET alone. a), b) & c) represent n=3 for these control experiments.

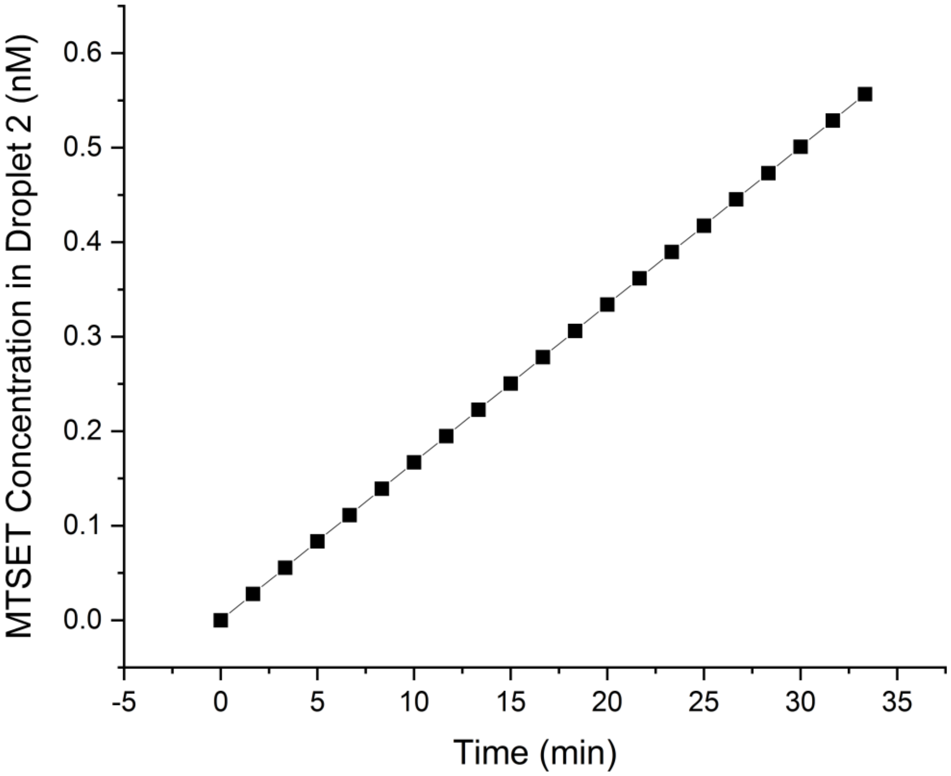

Supplementary Figure 10. Fick's first law approximation. Graph showing the results of a Fick's first law calculation of the change in concentration of MTSET in the second droplet of a DIB assuming MTSET cannot diffuse across the DIB, and assuming one subconducting MscL pore of 1.5nm diameter[3] and 4 nm thickness. In the time frame of the assay the concentration of MTSET in the second droplet is still orders of magnitude smaller than the first droplet, but the amount of MTSET in the second droplet is still sufficient to activate MscL as a trace number of molecules (5 molecules of MTSET) are needed to open the 5 subunits of a single MscL channel. It is noted that this approximation is assuming only one partially open channel, not taking into account the presence of multiple channels or diffusion across multiple subconductance states.

Supplementary References

1 Miller, D. M., Findlay, H. E., Ces, O., Templer, R. H. & Booth, P. J. Light-activated control of protein channel assembly mediated by membrane mechanics. *Nanotechnology* **27**, 494004 (2016).

2 Friddin, M. S., Morgan, H. & de Planque, M. R. Cell-free protein expression systems in microdroplets: Stabilization of interdroplet bilayers. *Biomicrofluidics* **7**, 014108 (2013).

3 Sukharev, S. I., Sigurdson, W. J., Kung, C. & Sachs, F. Energetic and spatial parameters for gating of the bacterial large conductance mechanosensitive channel, MscL. *J. Gen. Physiol.* **113**, 525–539 (1999).
